# An Account of a New Species of Swietenia (Mahogany); and of Experiments and Observations on Its Bark, Made with a View to Ascertain Its Powers, and to Compare Them with Those of Peruvian Bark, for Which It Is Proposed as a Substitute: Being an Abstract of a Paper on This Subject, Addressed to the Honourable Court of Directors of the United East-India Company

**Published:** 1795

**Authors:** William Roxburgh


### IX. *An Account of a new Species of Swietenia (Mahogany); and of Experiments and Obſervations on its Bark, made with a View to aſcertain its Powers, and to compare them with thoſe of Peruvian Bark, for which it is propoſed as a Subſtitute: Being an Abſtract of a Paper on this Subject, addreſſed to the Honourable Court of Directors of the United Eaſt-India Company. By* William Roxburgh, *M.D.*

THE ſpecies of Swietenia deſcribed in this paper, and which Dr. Roxburgh names *Swietenia Febrifuga* *, is a native of the mountainous part of the Rajamundry Circar, North of Samulcotah and Peddapore. It is a very large

* Dr. Andrew Duncan, junior, who has made this new ſpecies of Swietenia the ſubject of a very ingenious inaugural Diſſertation, gives a good reaſon for preferring, as a trivial name, the Hindoo appellation, *Soymida*, to one founded on its medicinal properties; ſimilar properties, he obſerves, being aſcribed by Dr. Wright (London Medical Journal, Vol. VIII. p, 286) to the mahogany tree of Jamaica (*Swietenia Mahagoni*), another ſpecies of the ſame genus.—Vide *Tentamen inaugurale de Swietenia Soymida*; *Auctore* Andrea Duncan. 8vo. Edinburgi, 1794. Editor.

large tree, known among the Hindoos by the name of *Soymida*, and flowers about the end of the cold or beginning of the hot feafon. Its feeds ripen in three or four months after.

Of this tree Dr. Roxburgh gives the following botanic defcription :

" TRUNK. Very ftraight, rifing to a great
" height, of a great thicknefs, and covered
" with a grey, fcabrous, cracked bark.

" BRANCHES. Numerous, the lower
" fpreading, the higher afcending, forming a
" very large fhady head.

" LEAVES. Alternate, about the extre-
" mities of the brachlets *(turiones)* abruptly
" feathered, about twelve inches long.

" LEAFLETS. Oppofite, very fhort, pe-

" tiolated, three or four pair, oval, obtufe, or

" end-nicked, the lower fide generally extend-

" ing a little further down on the petiolet than

" the upper; fmooth, fhining; from three to

" five inches long, and from two to three

" broad, the inferior fmalleft.

" PETIOLE. Round, fmooth, about nine
" to ten inches long.

" STIPULES none.

" PANICLE. Very large, terminal, dif-
fufe,

" fuſe, bearing great numbers of middle-ſized,

" white, inodorous flowers.

" PEDUNCLE and PEDICLES. Round

" and ſmooth.

" BRACTS. Very minute.

" CALYX. Below, five-leaved; LEAF-
" LETS. Oval, deciduous.

" COROL. Petals five, inverſe, egged,

" obtuſe, concave, expanding. NECTARY.

" Not quite half the length of the petals, a

" little bellied; mouth ten-toothed, teeth bi-

" fid (two-cleft).

" STAMEN. Filaments ten, very ſhort,

" inſerted juſt within the mouth of the nectary.

" ANTHERS. Oval.

" PISTIL. Germ conical. STYLE. Thick,
" tapering. STIGMA. Large, targetted, ſhut-
" ting up the mouth of the nectary.

" PERICARP. Capſule egged, large, five-

" celled, five-valved, valvelets gaping from

" the top.

" RECEPTACLE. In the centre, large,

" ſpongy, five-angled; angles ſharp and con-

" nected, with the ſutures of the capſule, be-

" tween them, deeply ſulcated.

" SEEDS. Many in each cell, imbricated,

" obliquely wedge-ſhaped, enlarged by a long

"membranaceous wing, inferted, at the upper
"point of the wing, into a long brown fpeck
"on the upper part of the excavations of the
"receptacle: all the reft of the receptacle is
"white."

The wood of this tree, we are told, is of a dull red colour, remarkably hard and heavy; and is reckoned, by the natives, by far the moft durable timber they know; on which account it is ufed for all the wood work in their temples.

The bark of the trunk and large branches, of large and middle-fized trees, is covered with a dark rufty-coloured coat, of about an eighth of an inch in thicknefs, which cracks in various directions, and fometimes peels off in irregular pieces, according to the directions of the cracks. Immediately under this is a very firm, but brittle coat, of about three-eighths of an inch in thicknefs. When firft cut, it is light-coloured; but on drying, or even expofure to the air for a few minutes, it turns to a reddifh brown. The inner lamina are thin, confifting of tough, lighter-coloured layers.

The bark of the younger branches is not cracked, is pretty fmooth, of a much lighter colour, colour, and has not the rufty coat above defcribed, but has often many blotches of various coloured lichen over it: it confifts wholly of the brown, folid, and inner layers.

The outer ruft-coloured layer of the trunk has but little tafte; the other two poffefs a little aromatic fmell, and their tafte is very bitter and aftringent, accompanied with fomething aromatic, but in a trifling degree. There is nothing difagreeable in the tafte, more than may be expected from a pure, fimple, ftrong bitter and aftringent united. The middle lamina are eafily reduced to a very fine rofe or light brown-coloured powder.

Cold water, in the courfe of an hour, our author obferves, acquired from this bark a deep but clear reddifh colour. The moft minute portion of a chalybeate (one drop of a folution of twenty grains of fal martis in an ounce of water) inftantly changed a much-diluted cold infufion to a deep purple, which, on ftanding, became darker and darker, with a reddifh tinge; and no decompofition took place till about the tenth day; the colouring matter then began to feparate, and fall to the bottom in black flakes, leaving the liquor almoft colourlefs. If the infufion was fome days (from four to thirty)

 old, old, the colour produced by the martial solution was as instantaneous as when fresh, and deeper; and at no period, up to thirty days, did it show the least tinge of green. Ten times the same quantity of the same martial solution, it seems, did not produce so great a change upon a similar infusion of the common pale Peruvian bark; and its effect on the latter was much slower. Its bitter qualities are also described as much more intense than those of the common sort of Peruvian bark.

The infusion, we are told, bears to be mixed in any proportion with spirits, without becoming turbid, or producing any kind of decomposition. The first decoction is considerably deeper-coloured than the infusion (which colour it retains in passing the filter), and possesses the same powers in a higher degree, but does not retain them so long, nor is it so pleasant to the taste. On standing any length of time with the chalybeate, the colour becomes pale, and is sooner decomposed than the cold infusion: on standing some days it lets fall a small quantity of a reddish, earthy fecula, which is intensely bitter and astringent; the superincumbent liquor becoming gradually clearer and clearer, and at the same time of a deeper red, much resembling the tincture. Lime-water added to the decoction, infusion, infuſion, or diluted tincture, darkened them conſiderably, and cauſed in all a copious depoſition of reddiſh brown fecula, which became purple coloured in twenty-four hours. The decoction, it is obſerved, gave the greateſt quantity of fecula. An infuſion of pale Peruvian bark, prepared in every reſpect the ſame as the infuſion of Swietenia bark, was treated with lime-water in the ſame manner, and formed a ſeparation, but in a much leſs degree.

Bark of *Melia Azadirachta* (Margoſa tree) treated exactly in the ſame manner, formed a ſeparation of a lighter-coloured fecula, in a much greater quantity than the common Peruvian bark, but much leſs than the Swietenia bark.

The clear reddiſh-coloured liquor, we are told, that floats over the precipitate cauſed by the addition of lime water, is void of aſtringency to the taſte, or has it only in a trifling degree; but for a farther proof, it ſeems, a chalybeate was employed, which did not in the leaſt darken this liquor; but a greeniſh tinge was produced, together with a further decompoſition and precipitation of a reddiſh fecula. This experiment, our author thinks, ſerves to ſhow that at leaſt the aſtringent part of the bark is entirely thrown down by lime-water; and he conſidered this as ſo intereſting a point, that he repeated the ſame experiment with this, as well as with other aſtringent barks, and found the reſult exactly the ſame.

The ſame chalybeate added to lime-water of the ſame ſtrength as that employed in the above-mentioned experiments, produced a ſmall, green cloud; the Swietenia bark infuſion thrown into this produced a muddineſs, and ſoon after, a copious precipitation of dirty-coloured fecula.

An infuſion of this bark in lime-water is deeper coloured than the plain infuſion, but poſſeſſes very little bitterneſs, and ſtill leſs aſtringency. A chalybeate added to this infuſion rendered its red colour a little deeper only, and no decompoſition took place: after ſtanding ſome time, the infuſion had no taſte of the lime-water.

From theſe experiments, Dr. Roxburgh conſiders lime-water as a very improper addition; and obſerves that, in this reſpect, they agree with thoſe made by Dr. Irving on the red and quilled Peruvian barks.

Vitriolic acid rendered the firſt decoction, or watery infuſion, paler; and, upon ſtanding, it became a little turbid, and let fall a ſmall quantity of a light-brown ſediment.

Vinegar had the ſame effect.

Mild, or cauſtic vegetable alkali, or mild foſſil foffil alkali, foon deepened and rendered brighter the cold watery infufion or decoction, nor did any decompofition take place in forty-eight hours.

Mild magnefia, fimply added, rendered the colour of the infufion paler, without fenfibly altering the tafte.

Alum has been at times fuccefsfully employed for the cure of intermitting fevers, and the analogy it bears to other tonics renders it a likely remedy. Our author was therefore defirous to try what would take place on adding it in a fmall quantity to infufions and decoctions of this bark. The addition, it feems, rendered their colour paler, and a little decompofition took place, with a precipitation of a fmall quantity of a light-brown fecula: to the tafte it increafed the aftringency without fenfibly diminifhing the bitter; but with alum they did not change their colour when a folution of green vitriol was added.

Eight ounces of the coarfe powder were boiled in ten pints of foft well-water to four pints; the refiduum was repeatedly boiled in frefh parcels of water, exactly in the fame manner for eleven times, when the liquor came off ftill much coloured, but taftelefs, and fhowed no figns of aftringency with the chalybeate; the tenth decoction excepted, which did fhow figns of aftringency, as it was darkened a little by it.

The frefh decoction of common Peruvian bark, made fimilarly, but in a fmaller quantity, ftruck flowly about as deep a colour with the fame chalybeate, as the fourth or fifth decoction of Swietenia bark did quickly.

As the eleventh decoction was taftelefs, although coloured, it was thrown away; the other ten had been regularly ftrained, while hot, and fuffered to ftand till perfectly cold, then poured off, clear from fediment; they were mixed, and evaporated to a hard extract, which weighed two ounces and three-quarters. The extract, when foft, was of a dark red colour, flavourlefs, fmooth, homogeneous, and unctuous when rubbed between the fingers and thumb. The tafte of the decoction was well preferved in this extract; the moft minute part of it, diffolved in water, ftruck a black colour with martial folution as quickly and as deep as the decoction itfelf, but the tafte was not fo ftrong as might be expected from that of the bark. This, our author thinks, might might perhaps be owing to the more fixed, inert parts, extracted by the long and repeated boilings (which lafted two days) being mixed in the mafs of extract. But this, he obferves, would not be the cafe, or but in a fmall degree, with one prepared from only one or two boilings. To determine this point, he boiled one ounce of the powdered bark in two pints of water, pretty brifkly, down to one pint; after the liquor was poured off, to the refiduum were added two other pints of water, and boiled in the fame manner. The decoctions were mixed, and evaporated to a dry extract, which weighed two drachms and a half, and was in tafte, &c. much as the former from ten coctions; the proportion of extract from two boilings is therefore, he obferves, nearly equal to that of ten: fo that, although the decoctions were highly coloured, and confiderably bitter and aftringent, even to the tenth, yet they could have contained but a fmall portion of the powerful qualities of the bark.

The refiduum, when perfectly dry, weighed four drachms and a half; and fpirit of wine being poured on it, though affifted at times with the heat of the fun for many days, extracted neither neither colour nor tafte, fo completely had the virtues of the bark been extracted by the water.

Dr. Roxburgh obferves that the dry extract imbibes much moifture when the weather is damp; fo much as to make it ftain the fingers, or any thing that touches it: that it melts readily in the mouth; is eafily foluble in water and in fpirits; and, like the decoction and tincture, bears to be mixed without decompofition. Thefe folutions and mixtures, we are told, refemble much the original decoction and tincture, and their mixtures, both in tafte and colour.

Should this ever become the valuable drug it promifes, it would be advifable, our author thinks, to have the extract prepared on or near the fpot where the trees grow. If this is done during the hot feafon, the evaporation, he obferves, might be effected by the heat of the fun and hot winds, which would certainly produce a much more elegant, efficacious extract than could poffibly be prepared in any other way or place, and would alfo preclude every idea or chance of its being fophifticated.

This bark, he finds, contains much mucilaginous matter, the cloth that the decoctions were were ftrained through, having become, when dry, ftiff as if ftarched. This, he thinks, may account for the decoctions remaining fo many days turbid, which is, no doubt, he adds, favourable for the action of the ftomach upon the bark. The late Dr. Fothergill, he obferves, recommended an addition of fome mucilage to decoctions of common bark, in order to keep them turbid, that the active parts might be kept more completely fufpended in the liquor *.

In the way of diftillation, this bark, it feems, yields nothing, not the fmalleft apparent quality, either with water or fpirits. In this refpect, Dr. Roxburgh thinks, it refembles exactly both the pale and red Peruvian barks, viz. in having its powers or virtues of a very fixed nature.

Rectified fpirit of wine extracts from the bark a clear, deep red tincture, poffeffing the aftringency of the watery infufion or decoction, and more of the bitter. If not too ftrong, it makes, we are told, one of the moft pleafant bitters we are in poffeffion of; and it bears to be diluted with water in any proportion, without decom-

* Med. Obf. and Inq. Vol. I. p. 321. 2d Edit. 8vo. London, 1758.

position, pofition, which renders it in many cafes the more defirable.

Four ounces of powdered bark were infufed, by our author, for eight days, in three pints of French brandy; thefe were poured off, and four pints more of the fame brandy added, which, after ftanding four days, were alfo poured off: both thefe infufions were mixed, and he drew off, by diftillation, a quantity of the fpirit, which (as before obferved) did not in the leaft partake of any of the qualities of the bark: the reft was gently evaporated to a dry extract, which weighed nine drachms. The extract itfelf was of a much darker colour than that procured by water, and was dried with more difficulty; but the tafte of the two extracts was much the fame. The refiduum was boiled in fix pints of water to two, and the decoction was found to be ftill pretty ftrong to the tafte, both in bitternefs and aftringency. This induced him to repeat the boiling, twice more, with frefh parcels of water; and the laft decoction, though weak, was ftill bitter, and fhowed figns of aftringency, with a martial folution. Thefe four decoctions were mixed and evaporated to a dry extract, weighing three drachms, which added to the fpirituous extract, tract, made in all twelve drachms, from four ounces of powdered bark, and agreed nearly with the quantity procured by water alone.

The antiseptic powers of this bark, according to our author's experiments, are not inferior to its bitter and astringent qualities; for watery infusions in open phials kept perfectly good for sixty days, without any tendency to fermentation, except a few air bubbles, which they discharged about the second day; indeed they acquired strength, we are told, as the colour produced at the end of that time (sixty days), by the addition of a chalybeate, was darker, and as instantaneous as at any prior period.

Sixty grains of the lean of raw mutton were preserved sweeter and firmer in an infusion of ten grains of this bark in four ounces of water, than an equal quantity of the same mutton in a similar infusion of pale quilled Peruvian bark. The flesh was tinged red by the infusion of Swietenia bark, and its fibres were firm and distinct at the end of twelve days; while that preserved in the Peruvian infusion was white, and its fibres softer, and infinitely more fetid.

Almost all the foregoing experiments, it is observed, obferved, were made firft with bark of the fmaller branches, and again with bark of the trunk of a large tree; the latter was evidently ftrongeft.

The feeds of this tree are defcribed as a ftrong, fimple, pleafant bitter, without any of the aftringent power. The leaves poffefs nearly if not all the aftringency of the bark, and a very large proportion of its bitter; but their tafte is faid to be not fo agreeable either in fubftance or in infufion.

From the foregoing analyfis, Dr. Roxburgh ventures to draw the following conclufions:

Firft. That the active parts of the bark of this fpecies of Swietenia are much more foluble than thofe of Peruvian bark, particularly in watery menftruums.

Secondly. That it contains a much larger proportion of active (bitter and aftringent) powers, than Peruvian bark.

Thirdly. That the watery preparations of this bark remain good much longer than fimilar preparations of Peruvian bark.

Fourthly. That the fpirituous and watery preparations bear being mixed in any proportion, without decompofition.

Fifthly. That the bark in powder, and its preparations, are much more antiseptic than Peruvian bark, or similar preparations of it.

Now, since this bark yields so much of its virtues to cold water, as to preserve flesh from corruption, in a hot climate, with the thermometer from 87° to 102°, it is reasonable, he contends, to suppose it will yield still more of its tonic and antiseptic virtues in the stomach, where it meets with the most powerful solvents: we have therefore, he thinks, much to expect from it in the cure of gangrene and other putrid diseases.

Bitters and astringents, in a separate state, our author observes, are considered as tonic remedies; but when found combined in the same substance, they become still more powerful: it is from these qualities, he contends, that the best judges allow the Peruvian bark to derive its virtues. On this point he quotes the authority of Dr. Cullen, who has remarked, "that the "recurrence of the paroxysms, in intermitting "and remitting fevers, depends on the recur-"rence of atony in the extremities of the arterial "system; hence they are prevented by such "tonic medicines as obviate this atony: a "great variety of astringents and simple bitters "have been found to answer that end, but "none, hitherto discovered, so effectually as the

"Peruvian

"Peruvian bark, on account, it is thought, of "its poffeffing thofe powers conjoined *."

The antifeptic qualities of Peruvian bark, our author obferves, are alfo great; hence its ufe in the cure of all febrile putrefcent diforders, attended with debility, putrid ulcers, &c.

From the evident qualities of this new bark, and from the fuccefsful experience he has had with it, in intermittent fevers †, &c. Dr. Roxburgh

* Treatife on the Materia Medica.

* Hiftories of feveral of thefe cafes have been communicated by Dr. Roxburgh to the College of Phyficians at Edinburgh, and an account of them is given by Dr. Duncan in the differtation referred to in a former note, together with the refults of feveral trials made with this bark, by his father, in the Clinical Ward of the Royal Infirmary at Edinburgh. We fhall take the liberty of tranfcribing this part of his work:

"Morbus, quo Roxburgius hunc corticem fæpiffimè adhi- "bendum curavit, febris quotidiana apud Cullenum nun- "cupatur. Rariùs ex toto, fed ex parte, et ad breve tantum- "modo tempus, remittens, periculofiffimus erat. Ægroti ferè "omnes hoc morbo correpti fuerant, dum incolebant iftos "montes ingentes, qui Indiæ peninfulam tranfcurrunt. Inter "hos montes fylvæ opacæ, denfa ferarum tecta, convalles pa- "ludofas, hominum generi peftiferas, ubique obumbrant. Se- "des eft indigenis etiam, confuetudine licet obfirmatis, infa- "lubris, advenis autem adeo perniciofa ut pauci, perpauci "quidem, quos dira neceffitas inter hos montes hiemare coege- "rit, morbo hoc atrociffimo immunes fint. Tali febre, tali "tempeftate burgh has every reafon to imagine it will prove equal, if not fuperior, to the Peruvian bark, for every purpofe for which that medicine is ufed.

Our

" tempeftate laborantium ne dimidiam quidem partem con-
" valefcere Roxburgius affimat.

" Cal. Junii, A. D. 1791. Indus annos natus viginti, " habitûs tenuis, nonnullis antè menfibus, dum prope montes " occupabatur, febre quotidianâ affectus erat. Corticem " Cinchonæ officinalis aliquantifper fine fructu affumpferat; " idcirco Roxburgius, et quia ipfe parvas corticis Soymidæ " quantitates impunè adhibuerat, ægro nihil à periculo ab- " horrenti grana viginti pulveris ex aquæ cyatho fumenda " præfcripfit. Duabus exinde horis, fcrupuli duo adhibiti " funt; et, poft fimile temporis intervallum, drachma. Cor- " tex ægro nequaquam ingratus erat, alvumque folvit. Æger, " cortice poftea ad drachmam, unaquâque intermiffione, af- " fumpto, triduo febre immunis erat.

" Pridie Iduûm Augufti, A. D. 1791, J— V— Lufi- " tanus*, annum agens quadragefinum quintum, ejufque duæ " filiæ, altera fex, altera tres annos nata, manferant aliquan- " diu, inter menfem proximè præteritum, intra montium ter- " minos; initioque menfis labentis, febre quotidianâ, quæ " nihil fermè quicquam remifit, affecti funt. Febre remit- " tente, femper alterâ quaque horâ fumebant aquæ ex cortice " Soymidæ* pater fefcunciam, filia major natu unciam, et " minor femunciam. Duos poft dies, à morbo valebant.

* " R. pulv. cort. Swiet. Soymidæ unciam unam,

" aquæ fontanæ libras duas.

" Mifceantur, et phialâ priùs agitatâ, modo præfcripto fumantur."

* Vide p. 148.

Our author next enumerates different species of Cinchona, viz.

First.

" Morbus, quo hi quatuor ægroti laborabant, partim ob " anni tempus, quo febre correpti sunt, atque partim ob " tempestatis siccitatem, solito levior erat; atque Roxbur- " gius, propter ægrorum debilitatem, neque evacuantia ad- " hibebat, nec intermissiones expectabat.

" xv. Cal. Sept. A. D. 1791, B— Lusitana, habitûs " infirmi, nonnullos dies, febre gravi, nunquam ex toto re- " mittente, laboraverat. Antimonium tartarisatum ex multâ " aquâ, partitis vicibus, usque ad vomitionem, adhibuit. " Postero die drachma corticis Soymidæ, in remissione mi- " nimè adhuc notabili, ter assumpta est. Intermissio proxima " plenior evasit, atque, ex corticis usu, biduo postea morbus " ipse, simulque diarrhœa quâ laboraverat ægra, cessarunt.

" Mense Septembris, A. D. 1791, J. E— decurio Euro- " pæus, annos natus quadraginta, febre remittente graviter " affectus est. Recessus principio ferè nulli, ex usu præpara- " torum ex antimonio notabiliores evaserunt; et æger, quan- " quam omni generi intemperantiæ deditus, cortice ter sin- " gulis intermissionibus adhibito, paucis diebus convaluit.

" vii. Cal. Sept. A. D. 1791, T. L— annos natus " octodecim, quosdam dies febre biliosâ laboraverat; cujus " recessus, etiam post antimonii tartarisati usum, parum " notabiles erant. Debilitate autem urgente, scrupuli duo " corticis Soymidæ, omni recessu, ter adhibebantur, et, ad " alvum solvendam, lixiva tartarisata.

" A cortice autem nihil proficiente, iii. Cal. decessum " est; atque medicamentis idoneis assumptis, febris prorsus

" fere,

First. *Cinchona Officinalis panicula brachiata*; to this species, he observes, belong the pale, quilled,

" fere, statis temporibus, intermisit. Soymida nunc iterum " adhibita, quatuordecim diebus, morbum penitus fugavit.

" Mense Septembris, A. D. 1791. S— nutrix lactans, " annos nata triginta quinque, febre quotidianâ correpta " est. Alvo, inter primam intermissionem, sodâ vitriolatâ " solutâ, morbus triduo cortice Soymidæ depulsus est; sed " lac interim fluere cessaverat.

" Mense Septembris, A. D. 1791. Indus, servus domes- " ticus †, febre singulariter intermittente ægrotavit. Sub " occasum solis, accessit febris gravis, quæ horâ nonâ ves- " pertinâ intermisit. Oriente autem sole, iterum accessit, " atque, horam circiter nonam ante meridiem, denuo inter- " mittens, ægrum viribus integrum reliquit. Exinde cor- " tice Soymidæ ter, singulis intermissionibus matutinis, ad " scrupulos duos adhibito, triduo morbus omnino evanuit.

" J— R— Europæus annum agens trigesimum, vitio pul- " monis multum debilitatus, ineunte Octobri febre quotidi- " anâ, cui erant accessiones vespertinæ, affectus est. Tertiâ " intermissione, duo corticis scrupuli bis adhibiti alvum " magnopere solverunt. Soymidâ nihilominus continuatâ, " æger quatuor diebus à febre valebat.

" Pridie Iduûm Decemb. R— miles Indicus, annos natus " triginta, febre quotidianâ tredecim dies, medicamentis " vernaculis nihil proficientibus, laboraverat. Intermissione " proximâ duo corticis Soymidæ scrupuli ex aquâ ter adhi- " biti alvum bis cierunt, morbumque levarunt. Cortex " repetitus ægro sanitatem restituit.

" Pridie Iduûm, Dec. A. D. 1791, L— miles Indicus,

† Vide p. 148.

 " annos quilled, and red barks, which the beſt judges imagine are from the ſame tree; the thickred

" annos natus viginti tres, antecedente die, febre quotidianâ " affectus eſt. Cortice ter ſingulis intermiſſionibus adhibito, " alvus ſoluta eſt, morbuſque mox remiſit.

" Pridie Iduûm Dec. S. N— miles Indicus, annos natus " quadraginta, IV. Non. Decemb. febre correptus erat. Nullis " hactenus medicamentis uſus, magis nunc magiſque debilis " evaſerat. Cortex in remiſſione ter adhibitus ventrem " ſolvit, triduoque morbum depulſit.

" Pridie Iduûm Dec. A. D. 1791. N— miles Indicus, " annum agens vigeſimum quintum, pridie febre quotidianâ " affectus erat. Cortex Soymidæ, ter in unaquaque inter- " miſſione adhibitus, alvum movit, atque morbum brevì ſu- " peravit.

" VIII. Cal. Martii, A. D. 1792. J. V— per biduum " febre iterum * laboraverat. Morbo autem duo acceſſus " totidemque remiſſiones quotidie erant, ejus inſtar paulò " ſuprà deſcriptæ †. Cortex Soymidæ, in matutinis inter- " miſſionibus, alterâ quaque horâ adhibitus, triduo febrem " curavit.

" Circiter medium Februarii, R— infector telæ xylinæ, " annos natus viginti quinque, laborans tumore hypogaſtrii " æquali, dolente, quem comitata eſt febris omni mane rece- " dens, atque alvus aſtricta, ad Roxburgium adductus eſt; " cui dixit, ſe duodecim antè dies affectum eſſe dolore circa " umbilicum torquente, qui uno alterove die gravis evaſit, " atque profundus, et, quaſi inter veſicæ urinariæ fundum " atque inteſtinum rectum, ſedem cepit; abdomen mox tu- " muiſſe, ipſumque toto corpore febricitâſſe; cauſam autem

* Vide p. 145. † Vide p. 147.

" ignorâſſe red sort being from the trunk, while the pale-quilled sort is from the branches, and from young

" ignorâsse malorum; multa denique remedia vernacula

" incassùm adhibuisse.

" Ei præcepit medicus, ut assumeret parvas lixiviæ tar-

" tarisatæ quantitates, donec superveniret catharsis, pro

" potu communi biberet aquam ex tamarindis coctam cum

" saccharo, et ut interea diætâ ex oryzâ famem tolleret.

" Alvo his exoneratâ, meliusculè se habere sensit æger;

" tumori autem nequaquam decrescenti, vesicatorium ad-

" motum est, alvusque lixivâ tartarisatâ et aquâ ex tama-

" rindis cum saccharo commistâ soluta est.

" Per noctem febris invaluit. Die autem, à curatione

" inceptâ, tertio alvus vehementer fluxit. Dejectiones

" purulentæ admodum erant, pessimè olentes, colore per-

" virides. Tumor statim subsedit.

" Æger maximè debilitatus, per noctem, graviter fe-

" bricitabat. Mane igitur, cùm primùm febris se remisis-

" set, ei pulvis ex Soymidæ cortice et lixivâ tartarisatâ com-

" positus adhibitus est, et, die progrediente, ter repetitus.

" His factis, alvus purulenta quædam quater dejecit. Hâc

" curatione triduo pòst à febre valebat, et, cortice nunc

" semel tantum in die adhibito, decem diebus domum re-

" diit sanus.

" Roxburgius unam tantum occasionem corticis Soymidæ

" contra gangrænam adhibendi nactus est. Viro dissoluto,

" per idem tempus lue Venereâ laboranti, super mediam

" tibiam ulcus erat. Cùm Soymidæ pulvis eius stomacho

" nigratus esset, extracto usus est, atque, expectatione ci-

" tiùs, morbo immunis evasit. Perhibet præterea Roxburyoung trees. The Spaniards themselves, he adds, employ the red sort.

Second.

" gius, Duffinum chirurgum valetudinarii Madrasiensis pri-

" marium hunc corticem contra istiusmodi mala maximo

" cum fructu adhibuisse.

" His memoratis, Roxburgius ingenuè fatetur insignem

" tempestatis siccitatem, hujus novæ Swieteniæ corticis

" usum feliciorem forsitan reddidisse. Notat præterea, cor-

" ticem primo die alvum plerumque movisse, postea autem

" nunquam, neque profectò, præter morbi curationem,

" ullos ex ejus usu effectus observâsse. Cur, ante corticis

" usum, non sæpiùs, ut mos plerisque est, vomitum et al-

" vum movisset, hanc rationem reddit, nempè ex regionis

" naturâ, ex victu, ex vitâ, atque ex religione, corpora

" Indis esse gracilia, nec plena, nec inflammationibus ob-

" noxia; atque remediis, quæ ante corticem adhiberi so-

" lent, febres, ut ille putat, in longum sæpe trahi, et iis,

" æque ac morbo fere ipso, ægrotos infirmari.

" Hæc uberiùs dixi atque fusiùs eò quòd ex his potissi-

" mum, quantum polleat hic cortex, apparet. His adduc-

" tus pater meus, cùm ægrotos nosocomio Edinburgensi

" curabat, atque discentibus de iis prælegebat, nova hujus

" corticis tentamina facere voluit. Hâc autem regione,

" cùm febris intermittens perrara sit, nobis nulla, quid pro-

" ficiat cortex noster, experiendi idonea satis occasio oblata

" est. Nonnullis autem ægrotis adhibita est.

" XIII. Cal. Jan. A. D. 1793. Joannes M'Kay, annum

" agens vigesimum tertium, priusquam in nosocomio recep-

" tus erat, duodecim dies febre, cujus accessiones altero

" quoque die redibant, laboraverat. Sed, cùm, ab initio

" horror

Second. *Cinchona Caribæa*; the Caribæan or

" horror et calor per idem tempus duravissent, sudor prorsùs defecisset, atque mala pectoris, coma, et torpor febrem comitata essent, hæc affectio minimè idonea, in quam novum medicamen tentaretur, videbatur. Cortex igitur Cinchonæ rubræ, per duodecim dies adhibitus est; cùm autem accessus post intervalla, licet valde dissimilia, adhuc redirent, ægro, ut Soymidæ drachmam alterâ quaque horâ sumeret, præscriptum est. Alvum torminibus magnopere movit, accessus autem proximus postremus erat. Convaluit.

" Jacobus Grant, annos natus viginti quinque, qui aliquandiu in nosocomio propter testis tumorem manserat, VIII Iduûm Junii, A. D. 1793, herbâ humidâ vesperi recumbens, frigore, gravi dyspnœa atque angustiæ in faucibus sensu, affectus est. Hæc facile æther vitriolico cesserunt, cortexque Cinchonæ, quo vires prostratas reficeret, adhibitus est. V. Iduûm iterum frigore, dyspnœâ, atque vomitione sanguinolentâ, correptus est. Quinto postea vespere horrores, intermittentis instar, accesserunt. Usum corticis Cinchonæ, quippe qui accessionibus nihil obstaret, intermisit medicus, pulveremque corticis Soymidæ, duplici autem quantitate, in ejus locum adhibuit. Hoc facto morbus nunquam postea rediit.

" Duabus adolescentulis, alteri à singulari affectione hystericâ, convalescentibus cortex Swieteniæ Soymidæ, ut corpora firmaret, si non cum utilitate saltem sine incommodo, adhibitus est.

" Vi insuper astrictoriâ pollere, satis constat è muliere annorum quadraginta sex, quæ leucorrhœâ laborabat.

or Jamaica bark of Dr. Wright *. This laſt, our author obſerves, poſſeſſes in a higher degree the bitter, but is very weak in the aſtringent power, and ought not to be depended on when the other is procurable.

Third. *Cinchona Sanctæ Luciæ, floribus paniculatis, glabris, laciniis linearibus tubo longioribus, ſtaminibus exertis, foliis ellipticis glabris;* Saint Lucia, or new bark. This is another ſort, which has been introduced into practice: but its being poſſeſſed of ſtrong emetic and purgative qualities, renders it, in our author's opinion, leſs eligible, particularly after the paſſages have been cleared. Theſe properties, he obſerves, the Jamaica bark does not poſſeſs; which eſtabliſhes a ſtriking difference.

Fourth. *Cinchona Corymbifera, foliis oblon-*

"Duobus ſenibus ventris fluxu affectis nihil profecit. Hi "autem, omnia, quæ alvum aſtringunt, experti, morbo "non levato, è noſocomio egreſſi ſunt.

"Cortex Soymidæ, ut multum, necne, contra putredi-"nem poſſet, appareret, quinque ægrotis typho putrido "laborantibus adhibitus eſt. Omnes convaluere. His "ventrem adeo non movit, ut, per totum morbum, alvum "aliis medicamentis ducere opus eſſet." *Vide* Duncan *Tentam. de Swietenia Soymida*, p. 41. et ſeq.—EDITOR.

* See Philoſ. Tranſact. Vol. LXVII. page 504; and London Medical Journal, Vol. VIII. page 239.

*gis, lanceolatis, corymbis axillaribus*; of Dr. Forſter; is a native of the South-Sea Iſlands: but of its virtues we know nothing more, than that he ſays, " it is like Peruvian bark, bitter " and aſtringent."

Fifth. *Cinchona Orixenſis, foliis oppoſitis, tomentoſis, ſtipulis interfoliaceis, ſemilanceolatis, floribus terminalibus, paniculatis, tomentoſis, capſula valvis contrariis à vertice dehiſcens*; of Dr. Roxburgh. The ſtructure of the capſule, he obſerves, forms the chief difference between this and Cinchona Officinalis, for the ſeeds are exactly as delineated by Gærtner, and the reſt of the definition correſponds with that given by Linnæus. It is a native of that chain of mountains which ſeparates the northern provinces, or circars, from the Mahrattah dominions immediately behind them. The bark of this ſpecies likewiſe is bitter and aſtringent.

Dr. Roxburgh has alſo found another new ſpecies of Swietenia, a middle-ſized tree, the wood of which is very heavy, cloſe-grained, and yellow; the bark likewiſe is yellow, and very bitter, but poſſeſſes much leſs aſtringency than that of the S. febrifuga, and its aſtringengy, he obſerves, is of a peculiar kind, for the colour produced, on an infuſion, with a martial ſolution, was a dark brown.

There

There is alſo the bark of another large tree, which, at the time of writing this account, he tells us, he had under examination, and which is likewiſe very bitter: the Hindoos call it *Wallurſe*. It will, he imagines, form a new genus in the claſs Decandria, and order Monogynia. Its eſſential characters are *calyx quinquefidus*, *petala quinque*, *nectarium duplex*, *exterius cylindricum ore decemfido*, *antheras gerens*, *interius annularium*, *baſin germinis cingens*, *bacca monoſperma*.

The bark of this tree, we are told, is in high repute as a medicine amongſt the Hindoo phyſicians; and gives name to a compound ſoft extract, called *Walluvoduſay*, which they employ in a variety of diſeaſes.

It alſo poſſeſſes powers of a very different nature; for, powdered and thrown into pools where there are fiſh, it ſoon intoxicates them to that degree, that they are eaſily taken with the hand.

Dr. Roxburgh obſerves that the bark of *Melia Azadirachta*, already taken notice of*, has frequently and ſucceſsfully been employed as a ſubſtitute for Peruvian bark, in the cure

* Page 133.

of remittents and intermittents; and that an infuſion or decoction of its leaves is alſo a good anthelmintic, and as ſuch employed by the Hindoos.

The bark of another large tree, which our author calls *Nauclea Daduga*, poſſeſſes alſo, he tells us, in a conſiderable degree, both the bitterneſs and aſtringency of Peruvian bark; and he thinks it is next in power to that of the Swietenia febrifuga. Although this tree differs widely in its flower from the hitherto known ſpecies of Cinchona, yet in its parts of fructification it agrees with them, it ſeems, almoſt exactly.

